# Combined transcervical radiofrequency ablation and hysteroscopic myomectomy: expanding treatment to diverse fibroid types

**DOI:** 10.1007/s00404-025-08127-y

**Published:** 2025-07-22

**Authors:** Elvin Piriyev, Angelika Dieter, Sven Schiermeier, Stefan Peter Renner, Thomas Römer

**Affiliations:** 1 Chair of Obstetrics and Gynecology, University Witten-Herdecke, Germany, Alfred-Herrhausen-Straße 50, 5093358455 Witten, Germany; 2 Department of Obstetrics and Gynecology, Academic Hospital Cologne Weyertal, Cologne, Germany; 3 Department of Gynecology and Obstetrics, Boeblingen Clinic, Hospital Sindelfingen-Böblingen, Böblingen, Germany; 4 University of Cologne, Cologne, Germany

**Keywords:** TFA, HSC, Fibroid, Operative hysteroscopy, AUB

## Abstract

**Introduction:**

Uterine fibroids are highly prevalent and often symptomatic, leading to abnormal uterine bleeding (AUB) and impaired quality of life. While hysteroscopic myomectomy (HSC) is the gold-standard treatment for submucosal fibroids, it is limited in addressing deeper lesions. Transcervical radiofrequency ablation (TFA) offers a minimally invasive alternative for intramural and transmural fibroids. This study evaluated the safety and effectiveness of combining TFA with HSC in a single session compared to HSC alone.

**Study design:**

We conducted a retrospective multicenter analysis of 127 women with symptomatic fibroids and AUB. Patients underwent either combined TFA + HSC (n = 75) or HSC alone (n = 52). Outcomes included intraoperative bleeding, complications, and symptom improvement.

**Results:**

The combined group treated a broader range of fibroid types (FIGO 0–6) and larger fibroids (mean size 2.86 cm vs. 2.23 cm; p = 0.0013). Intraoperative bleeding was significantly lower in the combined group (0% vs. 9.6%; p = 0.0102), with no increase in total complication rates (8% vs. 15%; p = 0.2512). Among patients with follow-up data, 85.1% reported symptom improvement after combined treatment.

**Conclusion:**

Combining TFA with hysteroscopic myomectomy is a safe and effective approach that expands the range of treatable fibroids, reduces intraoperative bleeding, and maintains high patient satisfaction. This integrated strategy offers advantages in tissue diagnosis, potential cost savings, and uterine preservation, making it a valuable addition to minimally invasive fibroid management.

## What does this study add to the clinical work

**Table:** 

Combining TFA with hysteroscopic myomectomy is a safe and effective strategy that expands the range of treatable fibroids and significantly reduces intraoperative bleeding, without increasing complication rates. This hybrid approach offers a valuable addition to minimally invasive fibroid management.	

## Introduction

Uterine fibroids (leiomyomas) affect up to 70% of women by the age of 50 and represent the most common benign gynecologic tumor worldwide. Although often asymptomatic, fibroids can produce a spectrum of clinical manifestations—including abnormal uterine bleeding (AUB), pelvic pain or pressure, bulk‐related symptoms, dyspareunia, and subfertility—that significantly impair quality of life and may necessitate surgical intervention [1, 2]. Traditional surgical management via abdominal myomectomy or hysterectomy remains effective but carries the risks associated with laparotomy and longer recovery times, and it may not be acceptable to women desiring future fertility or uterine preservation [3].

Minimally invasive, organ‐sparing therapies have therefore gained traction. Hysteroscopic fibroid resection (HSC) is considered the gold‐standard approach for submucosal (FIGO types 0–2) fibroids, allowing direct visualization and removal of intrauterine lesions with low complication rates and rapid recovery [4–6]. However, HSC is limited by its inability to address intramural or transmural fibroids (FIGO types 3–6) without laparoscopic or open access.

Transcervical radiofrequency ablation (TFA) has emerged as an incisionless modality capable of treating a broader fibroid spectrum (FIGO types 1–5) in an outpatient or short‐stay setting [7, 8]. The device combines intrauterine ultrasound imaging and volumetric, temperature‐controlled radiofrequency energy delivery in a single integrated instrument. Under real-time guidance, the fibroid is targeted, ablation and safety margins defined, and energy applied until a therapeutic temperature (105 °C) is reached. TFA has demonstrated substantial reductions in menstrual bleeding, durable symptom relief, and low reintervention rates at three years [9].

Despite their complementary scopes—HSC for intracavitary pathology and TFA for deeper lesions—the combined use of TFA followed by hysteroscopic myomectomy in a single session has been described only in small series [10]. Theoretically, pre-ablation may reduce intraoperative bleeding and facilitate resection of residual intramural components, while hysteroscopic removal addresses submucosal protrusion, optimizing symptom control.

To our knowledge, no large‐scale comparative studies have evaluated the safety and efficacy of this hybrid approach versus hysteroscopic resection alone. We therefore conducted a multicenter retrospective analysis of 127 women with symptomatic fibroids and AUB, comparing intra- and postoperative outcomes between those undergoing combined TFA + HSC and those receiving HSC only. Our primary aim was to determine whether combined treatment increases procedural risk; the secondary aim was to assess its impact on intraoperative bleeding, complications, and symptom improvement.

## Method and material

We conducted a retrospective analysis of patients treated for uterine fibroids at two German centers. Eligible patients presented with abnormal uterine bleeding (AUB) and sonographically or MRI-documented fibroids; those who were postmenopausal or whose bleeding was unrelated to fibroids were excluded. Patients were assigned to treatment groups based on fibroid characteristics. Those with both submucosal and intramural or subserosal fibroids were offered the combined TFA + HSC approach, while patients with only submucosal fibroids (FIGO types 0–2) were treated with operative hysteroscopy alone. This allocation was based on the differing anatomical access and capabilities of each technique. Participants were assigned to one of two groups:

- *Group 1 (Combined TFA + HSC)*: Patients received transcervical radiofrequency ablation (TFA) with the Sonata^®^ system immediately followed by bipolar operative hysteroscopic fibroid resection.
- *Group 2 (HSC alone)*: Patients underwent bipolar operative hysteroscopic fibroid resection without prior TFA.

Data for the TFA + HSC group were collected between January 2020 and October 2024. For the HSC-only group, data were randomly collected from patient records during the year 2022.

### Transcervical ablation procedure (Group 1 only)

After standard cervical dilation, the TFA device was introduced and its central spike (“introducer”) secured the target fibroid. Under real-time intrauterine ultrasound guidance, ablation and safety margins were defined, electrodes deployed, and energy applied once a tissue temperature of 105 °C was achieved.

### Operative hysteroscopy (Both groups)

Bipolar hysteroscopic resection of fibroids was performed in a routine fashion following device guidelines.

All surgeries were performed by experienced endoscopic surgeons, certified by the German Society for Gynecological Endoscopy at minimal invasive surgery (MIS) Levels II and III. We recorded intraoperative and postoperative complications, overall morbidity, and—for Group 1—changes in bleeding symptoms. Follow-up assessments were conducted via in-person postoperative visits or telephone contact. Follow-up data were available only for the TFA + HSC group, as these patients were routinely seen in a dedicated consultation hour established for TFA cases. No systematic follow-up was available for the HSC-only group due to the absence of a comparable postoperative consultation structure.

### Statistical analysis

Continuous variables are presented as mean ± standard deviation and were compared using independent-samples t-tests. Categorical variables were analyzed with Fisher’s exact test. All analyses were performed using SPSS software (Version 25.0, SPSS Inc., Chicago, IL, USA) and statistical significance was set at p < 0.05.

## Results

A total of 127 patients were included in the study: 75 in Group 1 (Sonata treatment followed by operative hysteroscopy) and 52 in Group 2 (operative hysteroscopy only). Baseline characteristics were comparable between the groups (Table 1). The mean age was 41.24 ± 6.51 years (range 28–56) in Group 1 and 40.23 ± 7.64 years (range 23–54) in Group 2 (*p* = 0.4250). Similarly, the mean BMI was 25.01 ± 4.55 kg/m^2^ (range 18.6–38.9) in Group 1 and 25.28 ± 6.06 kg/m^2^ (range 19.0–51.4) in Group 2 (*p* = 0.7748).

**Table 1:** Demographic and Clinical Characteristics of the Study Groups

Parameter	Group 1 Sonata + Operative hysteroscopy (n = 75)	Group 2 operative hysteroscopy only (n = 52)	p-value
Age (years)	41.24 ± 6.51 (28–56)	40.23 ± 7.64 (23–54)	0.4250
BMI (kg/m^2^)	25.01 ± 4.55 (18.6–38.9)	25.28 ± 6.06 (19.0–51.4)	0.7748
AUB—total cases	75 (100%)	52 (100%)	1.0000
Hypermenorrhea	68 (90.7%)	48 (92,3%)	1.0000
Menorrhagia	7 (9.3%)	4 (7,7%)	0.7599
No. of fibroids per patient			
1 fibroid	25 (33.3%)	47 (90,4%)	**0.0001**
2–4 fibroids	31 (41.3%)	5 (9,6%)	**0.0001**
> 4 fibroids	12 (16.0%)	—	—
Total fibroids treated	206	57	—
Fibroid size (cm)	2.86 ± 1.34 (0.7–7.0)	2.23 ± 1.09 (0.8–6.0)	**0.0013**
FIGO classification			—
FIGO 0–2	76 fibroids	57 fibroids	**0.0001**
FIGO 3–4	49 fibroids	—	—
FIGO 5–6	25 fibroids	—	—
FIGO 2–5	42 fibroids	—	—
Ablation time	8 min 44 s ± 7 min 56 s (1–37 min 48 s)	Not applicable	—
Follow-up duration (months)	7.00 ± 4.65 (3–24)	Not reported	—

All patients presented with abnormal uterine bleeding (AUB), primarily due to hypermenorrhea (90.7% in Group 1 vs. 92.3% in Group 2). Menorrhagia was reported in 9.3% of patients in Group 1 and 7.7% in Group 2.

The distribution of fibroid burden differed significantly between groups. In Group 1, 33.3% of patients had a single fibroid, 41.3% had 2–4 fibroids, and 16.0% had more than four fibroids. In contrast, 90.4% of Group 2 patients had only one fibroid, and 9.6% had 2–4 fibroids. Group 1 had a higher total number of fibroids treated (n = 206) compared to Group 2 (n = 57). The mean fibroid size was significantly larger in Group 1 (2.86 ± 1.34 cm; range 0.7–7.0 cm) than in Group 2 (2.23 ± 1.09 cm; range 0.8–6.0 cm) (*p* = 0.0013).

In terms of fibroid classification (FIGO), Group 1 included a broader range: 76 fibroids were classified as FIGO 0–2, 49 as FIGO 3–4, 25 as FIGO 5–6, and 42 as FIGO 2–5. In Group 2, only FIGO 0–2 fibroids (n = 57) were treated.

The mean ablation time in Group 1 was 8 min and 44 s ± 7 min and 56 s (range: 1 min to 37 min and 48 s). The mean follow-up duration in Group 1 was 7.00 ± 4.65 months (range 3–24 months). Follow-up data for Group 2 were not systematically recorded.

Regarding complications (Table 2), intraoperative bleeding occurred in 5 patients (9.6%) in Group 2, while no such cases were recorded in Group 1, demonstrating a statistically significant difference (*p* = 0.0102). Other complications occurred infrequently and did not differ significantly between groups: endometritis (1.3% in Group 1 vs. 0% in Group 2; *p* = 1.0000), uterine perforation (2.7% vs. 0%; *p* = 0.5126), intraoperative hemodynamic instability (1.3% vs. 1.9%; *p* = 1.0000), postoperative hematometra (0% vs. 1.9%; *p* = 0.4094), and fibroid expulsion (2.7% vs. 1.9%; *p* = 1.0000). The total complication rate was 8.0% in Group 1 and 15.0% in Group 2 (*p* = 0.2512).

**Table 2:** Comparison of Complication Rates in Combined TFA and Operative Hysteroscopy Versus Operative Hysteroscopy Alone

Group 1	N	Group 2	N	P value
Endometritis	1 (1,3%)	Endometritis	0	1.0000
Perforation	2 (2,7%)	Perforation	0	0.5126
Intraoperative bleeding	0	Intraoperative bleeding	5 (9,6%)	**0.0102**
Intraoperative hemodynamic instability	1 (1,3%)	Intraoperative hemodynamic instability	1 (1,9%)	1.0000
Postoperative hematometra	0	Postoperative hematometra	1 (1,9%)	0.4094
Postoperative fibroid expulsion	2 (2,7%)	Postoperative fibroid expulsion	1 (1,9%)	1.0000
Total	6 (8%)	Total	8 (15%)	0.2512

Among the 47 patients with available follow-up data in Group 1, clinical improvement was reported in 40 cases (85.1%). Two patients experienced fibroid expulsion, and one case of STUMP (smooth muscle tumor of uncertain malignant potential) resulted in subsequent hysterectomy.

The sample size calculation was based on the reported prevalence of intraoperative bleeding during hysteroscopic fibroid resection [11], estimated at 4%. Anticipating a 2% reduction in the bleeding rate following pre-treatment with transcervical fibroid ablation (TFA), and setting the significance level (α) at 0.05 with a power of 80%, the required sample size was calculated to be 1,139 patients per group. As the actual sample size did not meet this target, a post hoc power analysis was conducted according to the bleeding risks, revealing an achieved power of 81,8%, which is considered acceptable.

## Discussion

Our study demonstrated that combining transcervical fibroid ablation (TFA) with hysteroscopic myomectomy (HSC) allowed for treatment of a broader range of fibroid types (FIGO 0–6), significantly reduced intraoperative bleeding, and maintained a similar general complication profile compared to HSC alone. Notably, no intraoperative bleeding events occurred in the combined group, despite a higher fibroid burden and larger average fibroid size. These findings suggest that the combined approach offers both safety and enhanced procedural efficacy. In light of these results, we now compare our data to the existing literature on TFA and hysteroscopic myomectomy.

TFA has been established as a safe and effective uterus-sparing treatment for symptomatic fibroids [12]. Large trials (FAST-EU, SONATA) have shown marked improvements in bleeding and quality of life after TFA, with low reintervention rates [12, 13]. Hysteroscopic myomectomy (HSC) is likewise a proven treatment for submucosal fibroids, but its indications are traditionally limited to FIGO types 0–2 and it often requires staged procedures for large or deep lesions. For example, Emanuel et al. reported that ~ 17% of women undergoing hysteroscopic myomectomy needed multiple operations to complete fibroid removal [12, 14]. In contrast, the combined approach of initial TFA followed by hysteroscopic resection leverages the strengths of both techniques. These results align with prior reports—a recent case series of 21 patients found that transcervical RFA could be safely combined with operative hysteroscopy without added morbidity, with all patients satisfied and most reporting symptom improvement [10]. Similarly, TFA on its own addresses fibroids inaccessible to hysteroscopy (FIGO 3–6), whereas hysteroscopy effectively treats submucosal fibroids [10]. By comparing our multicenter retrospective outcomes to the literature, we confirm that the combined modality is at least as safe as either procedure alone and provides complementary benefits.

**Fig. 1:**
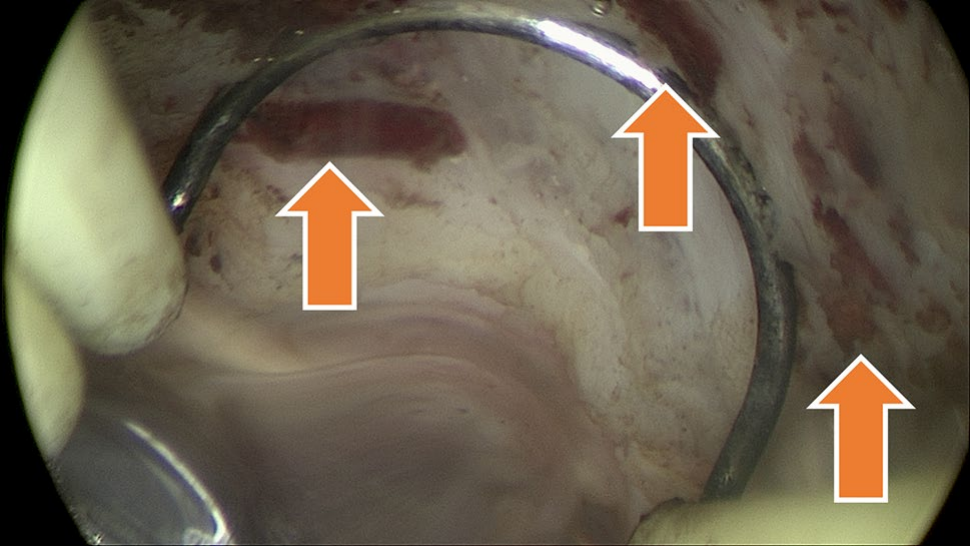
Fibroid resection performed immediately following transcervical fibroid ablation. The arrows highlight the thrombosed vessels within the fibroid

A key finding of our study was significantly lower intraoperative blood loss in the combined TFA + HSC group compared to hysteroscopic myomectomy alone. This is plausible given that radiofrequency energy induces immediate coagulation of fibroid vasculature (Fig. 1). Our findings suggest that pre-ablating fibroids with the TFA system appears to reduce hemorrhage during subsequent resection. This bleeding reduction has practical advantages for patient safety. Importantly, despite adding an extra step (the ablation), we observed no increase in operative time or complications, consistent with our previous results, in which we reported no difference in short-term morbidity between combined and standalone procedures [10].

Another major advantage of the combined strategy is the ability to obtain tissue for histopathology. Hysteroscopic resection yields fibroid fragments that can be examined microscopically, providing diagnostic confirmation. Although uterine sarcoma is rare, the practice of biopsy before TFA treatment (as in clinical trials) underscores the importance of histology. The combined approach ensures a specimen is available post-ablation, giving surgeons and patients greater reassurance. Thus, beyond bleeding control, the combined method adds diagnostic certainty—a benefit not realized with ablation-only techniques.

Sonata TFA can treat all non-pedunculated fibroids, effectively reaching intramural and transmural lesions (FIGO types 1–6) that are out of reach for hysteroscopy [7]. Hysteroscopic myomectomy, on the other hand, excels at removing submucosal fibroids (types 0–2) under direct visualization. By pairing these methods in one session, the combined approach achieves an unprecedented breadth of coverage. As noted in the literature, TFA “treats a wide spectrum of fibroids, including those not accessible by surgical hysteroscopy (FIGO 3,4,5,6)” [10]. Therefore, in a single operative setting we can address fibroids ranging from intracavitary (type 0) through deep intramural (type 3–4) and those with serosal extension (type 5–6). This integrated strategy reduces the need for multi-stage interventions. For instance, large type 2 fibroids often require interval resection after partial hysteroscopic removal; with ablation first, we can shrink or necrose the deep component and then complete resection immediately, rather than waiting for extrusion. In summary, combining TFA and HSC effectively bridges the FIGO gap: all fibroids from type 0 up through type 6 can be managed in one procedure, maximizing fibroid clearance and symptom relief without resorting to multiple surgeries. Importantly, the addition of TFA did not lead to an increased risk of severe intraoperative events or postoperative morbidity, even though it involved the treatment of a broader spectrum of fibroid types (including intramural and transmural fibroids not accessible by hysteroscopy alone). The integration of real-time intrauterine ultrasound guidance in TFA likely contributed to the safe delineation of ablation zones and avoidance of thermal injury to surrounding tissues.

Both TFA and hysteroscopic myomectomy are uterine-conserving therapies. Our combined protocol thus remains consistent with fertility preservation. Indeed, recent reports of TFA-treated women show encouraging pregnancy outcomes [15, 16]. In one series 72 women achieved 89 pregnancies (55 deliveries) post-TFA, with normal term outcomes and no cases of uterine rupture or placenta accreta [16]. These data suggest that TFA does not adversely affect the uterine wall. Likewise, hysteroscopic resection of fibroids is the standard fertility-preserving option for submucosal lesions. By offering both in one setting, we treat pathology effectively while maintaining reproductive potential.

Patient satisfaction is also an important outcome. In our previous series, nearly all combined-treatment patients reported symptom improvement and satisfaction with the procedure [10]. In the presented study, 85% of patients treated with the combined approach reported symptoms improvement. This mirrors high patient satisfaction seen after TFA in trials (with durable relief of bleeding and bulk symptoms) and after hysteroscopic fibroid removal [5, 12, 13]. The minimally invasive, incisionless nature of the procedures contributes to rapid recovery and high acceptability.

There are potential economic and logistical benefits. Performing TFA and hysteroscopic resection in a single session can obviate the need for second-look or staged surgeries. In contrast, multiple operations—as occur in up to ~ 17% of standalone hysteroscopic resections—incur extra facility and anesthesia costs [14]. By consolidating care into one day, the combined approach saves operating room time and patient time. In analogous studies, laparoscopic RFA required ~ 35% fewer surgical instruments than laparoscopic myomectomy, and allowed a faster return to work (average 11 vs 18 days) [15]. Although direct cost comparisons for transcervical procedures are limited, the outpatient feasibility of TFA (designed to be done as a same-day procedure) and shorter recovery suggest overall cost-effectiveness. Fewer hospital resources (and no overnight stays) and less time off work further enhance the value of the combined approach.

## Limitations

Several important limitations temper our conclusions. First, this was a retrospective, non-randomized study. Second, follow-up data were incomplete: long-term symptom and quality-of-life outcomes were assessed in the combined group but not systematically collected for the HSC-only group. Third, our assessment of bleeding and other intraoperative outcomes was based on operative records, which may have subjective elements. Future prospective, controlled studies with standardized follow-up would help to validate these findings.

## Conclusion

In summary, our multicenter experience suggests that combining TFA with hysteroscopic myomectomy is safe and effective, expanding the range of treatable fibroids while significantly reducing blood loss. Compared to hysteroscopic resection alone, the combined approach treats a broader spectrum of FIGO types (0–6) in one session and yields the added advantages of tissue diagnosis and potential cost savings, without compromising uterine preservation. These benefits—balanced against the limitations noted—indicate that the combined modality may be a valuable addition to the fibroid treatment options.

## Data Availability

No datasets were generated or analysed during the current study.
